# Disinfection of contaminated metal implants with an Er:YAG laser

**DOI:** 10.1002/jor.24662

**Published:** 2020-03-24

**Authors:** Lukas K. Kriechbaumer, Wolfgang Happak, Klaus Distelmaier, Gerhild Thalhammer, Georg Kaiser, Sylvia Kugler, Yulong Tan, Matthias Leonhard, Beata Zatorska, Elisabeth Presterl, Sylvia Nürnberger

**Affiliations:** ^1^ University Clinic of Orthopaedics and Traumatology Paracelsus Medical University Salzburg Austria; ^2^ Division of Trauma‐Surgery, Department of Orthopedics and Trauma‐Surgery Medical University of Vienna Vienna Austria; ^3^ Division of Plastic and Reconstructive Surgery, Department of Surgery Medical University of Vienna Vienna Austria; ^4^ Division of Cardiology, Department of Internal Medicine II Medical University of Vienna Vienna Austria; ^5^ Department of Dermatology, Division of Immunology Allergy and Infectious Diseases Medical University of Vienna Vienna Austria; ^6^ Department of Otorhinolaryngology and Head and Neck Surgery Medical University of Vienna Vienna Austria; ^7^ Department of Infection Control and Hospital Epidemiology Medical University of Vienna Vienna Austria

**Keywords:** biofilm removal, DAIR, Er:YAG laser, implant‐related infection, laser disinfection, pin‐tract infection

## Abstract

Infections related to orthopedic procedures are considered particularly severe when implantation materials are used, because effective treatments for biofilm removal are lacking. In this study, the relatively new approach for infection control by using an erbium:yttrium‐aluminum‐garnet (Er:YAG) laser was tested. This laser vaporizes all water containing cells in a very effective, precise, and predictable manner and results in only minimal thermal damage. For preliminary testing, 42 steel plates and 42 pins were seeded with mixed cultures. First, the minimally necessary laser energy for biofilm removal was determined. Subsequently, the effectiveness of biofilm removal with the Er:YAG laser and the cleansing of the metal implants with octenidine‐soaked gauze was compared. Then, we compared the effectiveness of biofilm removal on 207 steel pins from 41 patients directly after explantation. Sonication and scanning electron microscopy were used for analysis. Laser fluences exceeding 2.8  J/cm^2^ caused a complete extinction of all living cells by a single‐laser impulse. Cleansing with octenidine‐soaked gauze and irradiation with the Er:YAG laser are both thoroughly effective when applied to seeded pins. In contrast, when explanted pins with fully developed biofilms were analyzed, we found a significant advantage of the laser procedure. The Er:YAG laser offers a secure, complete, and nontoxic eradication of all kinds of pathogens from metal implants without damaging the implant and without the possible development of resistance. The precise noncontact removal of adjacent tissue is a decisive advantage over conventional disinfectants. Therefore, laser irradiation could become a valuable method in every debridement, antibiotics, and implant retention procedure.

## 1 INTRODUCTION

Infections represent the most common complication after surgical procedures and are of special concern when alloplastic implants are used. To date, the removal of the infected implant is frequently the ultimate therapeutic option. However, this procedure is associated with additional operations and commonly an inferior outcome for the affected patient.

Implant‐related infections are usually caused by microorganisms that form biofilms.^1^, ^2^ Within biofilms, microorganisms are enclosed in a polymeric matrix and develop into complex communities, resembling multicellular organisms.^3^ The biofilm shields the bacteria from host immune responses and from antimicrobial agents or antibiotics. Reports in the literature indicate that 500 to 5000 times higher levels of antibiotics are needed to achieve the same antimicrobial effects on biofilm bacteria than are needed for planktonic bacteria.^4^, ^5^, ^6^, ^7^

To prevent implant‐related infections, several alternative strategies have been tested in the last decade. These studies have investigated the bactericidal effects of silver and antibiotics used as coatings for metal implants^8^, ^9^, ^10^, ^11^, ^12^, ^13^ but showed elevated blood levels of silver ions,^8^ clinical failure,^10^ and even the development of bacterial resistance.^11^, ^12^, ^13^, ^14^

A relatively new approach to the treatment of microbial infections has been developed by dental surgeons, who use laser irradiation (erbium:yttrium‐aluminum‐garnet [Er:YAG] or Nd:YAG lasers) to remove biofilms from teeth and implants in the case of periodontitis and peri implantitis. These procedures look quite promising and could be converted to traumatologic and orthopedic settings. The Er:YAG laser emits infrared light at a wavelength of 2.94 µm which corresponds to the absorption maximum of water. Due to the high absorption of this wavelength in water, the irradiant energy of the laser pulse evaporates a relatively small tissue volume, which leads to microexplosions and an effective superficial tissue ablation (~60 µm/pulse) with minimal thermal damage (<10 µm/pulse). The Er:YAG laser is already widely used for surgical incision/excision, cutting ablation, vaporization, and coagulation of soft and hard tissue. Orthopedic surgeons could also profit from developments in laser technology. The perspective of having one more arrow in the quiver in a debridement, antibiotics, and implant retention (DAIR) procedure, where osteosynthesis equipment or prosthetic components that are fixed to bone are left in place, would be extremely desirable.

The aim of this study is to (a) evaluate if the Er:YAG laser disinfection of metal implants can be implemented in an orthopedic or traumatologic setting; (b) identify the correct laser parameters for a complete and secure biofilm removal; (c) compare if laser disinfection offers an advantage over other established disinfection methods; and (d) to identify the most common pathogens in pin‐site infection and to assess how reliable swab cultures are compared to implant sonication.

## 2 METHODS

Level of Evidence: II‐a.

We used an in vitro approach with seeded metal implants as well as an ex vivo approach with contaminated half pins from extracted external fixators. External fixators gained wide acceptance in the treatment of open and juvenile fractures, in polytraumatised patients or deformity correction.^15^ They were used for investigation because its pin sites are especially prone to infection, since the permanent skin wound facilitates the biofilm formation around the metal surface. Reported rates of pin‐site infection vary widely in the literature, ranging from virtually zero to considerably over 50%, and may cause local infections and pin loosening but also osteomyelitis or sepsis.^16^, ^17^, ^18^, ^19^, ^20^, ^21^

For laser irradiation, we used a Burane Er:YAG laser (Wave Light, Germany) with a maximum energy of 2000 mJ and a maximum power of 20 W. The laser beam was used in the slightly defocused mode (spot size 3 mm diameter), applying 1600 mJ at a frequency of 8 Hz. The fluence of the ablative pulses was 22.8 J/cm^2^. These parameters are the result of our preliminary findings, the rise in temperature within the pins, and the well‐known predictable ability of tissue removal of about 2.5 μm/pulse/J/cm^2^ and the collateral thermal tissue damage of about 20 µm.^22^, ^23^

This study was approved by the local Ethics Committee of the Medical University of Vienna and adhered to the Declaration of Helsinki. All patients provided written informed consent.

### 2.1 In vitro experiments using Er:YAG laser disinfection

We used a polymicrobial biofilm in vitro model. For in vitro biofilm formation, 42 sterile steel plates (15 × 15 mm, sectioned from Angled Blade Plates; DePuy Synthes, Switzerland) and 42 sterile steel pins (250 × 5 mm, sectioned from Apex Self‐drilling Half Pins; Stryker Trauma AG, Switzerland) were coated with fetal bovine serum for 24 h at 37°C and then seeded with mixed cultures of two bacterial species (*Staphylococcus epidermidis* [DSM 20044] and *Staphylococcus aureus* [ATCC25923]) and one yeast species (*Candida albicans,* ATCC 10231) with a concentration of 10^7^ colony‐forming units (CFUs). Then, they were covered with tryptic soy broth (TSB) medium and cultivated for 2 weeks at 100 rpm in well titer plates with daily replenishment of the growth medium.

For assessment of the minimal laser energy necessary for complete biofilm removal with a single‐laser exposure, 28 cultivated plates were irradiated all over line‐by‐line with an overlap of approximately 10% at constant spot diameter but with increasing pulse energies (0.2‐2 J). These plates were sonicated in 5 mL phosphate‐buffered saline and the total CFUs of residual viable yeasts and microbes were counted by plating out a series of dilutions on TSB agar plates incubated for 24 hours at ambient air conditions at 37°C.

For scanning electron microscopy (SEM) examination, the centers of another 14 seeded plates were irradiated with a single‐laser impulse also using increasing pulse energies (0.2‐2 J). These specimens were fixed in formaldehyde 7.5% and prepared for SEM according to a standard procedure.^24^

Fourty‐two in vitro‐seeded steel pins were divided into four groups and treated with the Er:YAG laser (1.6 J, spot size of 3 mm) either vertically (90°) or inclined (45°), cleaned mechanically with gauze soaked in octenidine (Octenisept, 0.1 g octenidine dihydrochloride and 2 g 2‐phenoxyethanol, Schülke&Mayr Ges. m. b. H., Vienna, Austria) or kept untreated as control. Octenidine was used because of its broad spectrum antimicrobial effects against both Gram‐positive and Gram‐negative bacteria and fungi,^25^, ^26^ the resistance to blood and albumin,^27^ its constant efficiency in the presence of organic matter,^28^ and the low allergic potential.^26^

For microbiological assessment, the laser was applied all over, whereas for SEM investigation, it was applied just in a single line along the pin to see the difference in the original and the lasered biofilm area.

Because potential collateral thermal damage is of concern in a surgical setting, additional sterile steel pins (diameters 3, 4, and 5 mm) were used to measure the rise of temperature during the laser irradiation process at different laser energies with an infrared thermometer (Exergen Temporal Scanner Infrared‐TAT 5000; Exergen, Watertown, MA). In all laser experiments, no cooling devices (air, air‐water spray, or flushing) were used.

We also tested the effect of the Er:YAG laser on 14 titanium plates (sectioned from PHILOS plates; DePuy Synthes) and observed that in contrast to steel implants, a macroscopic change of color occurred when very high‐power settings (203 J/cm^2^) were used. These high‐power settings were achieved by decreasing the spot diameter to 1 mm. This change of color is a well‐known process for titanium and titanium alloys and is called an annealing process. This process results from the application of laser light to the surface, which causes local heating and oxidation of the metal, whereby oxygen is absorbed from the air.^29^, ^30^

We investigated if this thin oxide layer modifies the surface topography, the adhesion ability, or growth kinetics of microorganisms. Therefore, the titanium plates were irradiated with the Er:YAG laser at intermediate (22.8 J/cm^2^) or very high (203 J/cm^2^) power settings or kept untreated as control, and, subsequently, seeded with two different biofilm building bacteria (S. aureus and S. epidermidis) for 3 weeks. We evaluated the titanium surface morphology after annealing and surveyed the bacterial attachment and proliferation to this surface by SEM and sonication.

### 2.2 Ex vivo experiments using Er:YAG laser disinfection

**Figure 1:**
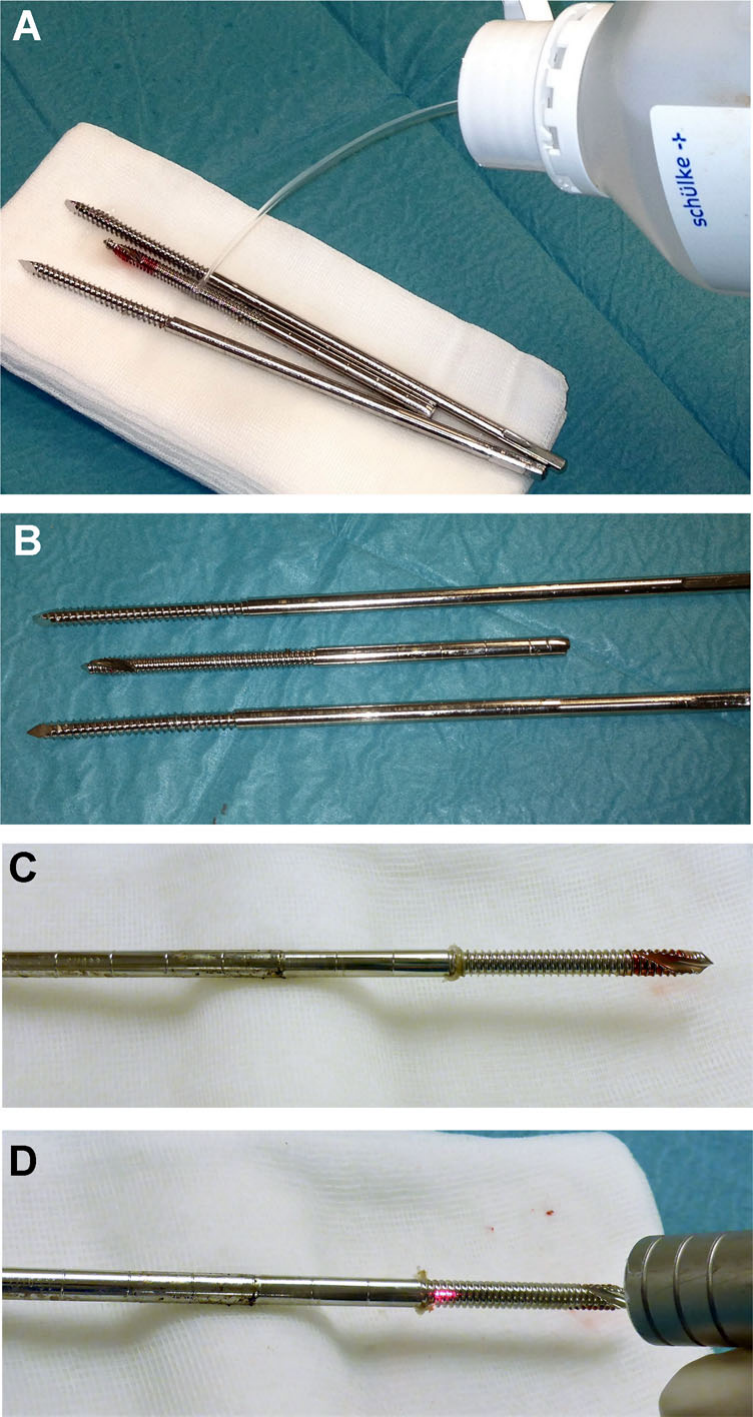
Treatment of the extracted pins. A and B, Wiping with sterile gauze saturated with octenidine and exposed to the germicide for the appropriate minimum contact time of 3 minutes. C and D, Irradiation with the laser. The red spot serves as the guiding beam for the otherwise invisible infrared Er:YAG laser beam. Er:YAG, erbium:yttrium aluminum garnet [Color figure can be viewed at wileyonlinelibrary.com]

Patients with fractures who had been treated between 2013 and 2017 at the Department of Trauma Surgery at the Medical University of Vienna with external fixators for at least 2 weeks were enrolled into this prospective study. Altogether, 207 steel pins were extracted and treated in the same way as the in vitro‐cultivated pins: group A with Er:YAG laser exposure at 1.6 J and 3 mm spot size (n = 69 pins), group B with octenidine wiping (n = 69 pins) (Figure 1), and group C was kept untreated as the control (n = 69 pins). After the group‐specific treatment, the pins were placed in sterile plastic containers and Ringer's solution was added before the containers were subjected to sonication according to the method described by Trampuz et al.^31^ We used a BactoSonic Biofilm‐sonication bath (BANDELIN electronic GmbH & Co. KG, Germany) with 35 kHz for 1 minute and an Eppendorf Centrifuge 5810 R (Eppendorf AG, Germany) at 4000 rpm for 5 minutes.

In addition, we used SEM examination to visualize the induced biofilm removal. Therefore, the laser was applied just in a single line along the pin.

On the day when the external fixator was removed, the pin sites were clinically evaluated according to the pin grading system proposed by Clint et al,^32^ which is based on three variables (erythema, discharge, and pain) and comprises three grades, named “good,” “bad,” and “ugly.” Independent and experienced surgeons performed this evaluation and they divided the extracted half pins into three groups with the only constraints being that the overall amount of metal and pins proximally and distally to the fracture had to be evenly distributed. In addition, they took swab cultures from the pin sites of the control group.

### 2.3 Statistics

Discrete data were described by absolute and relative frequencies and compared between groups using *χ* ^2^ tests. Continuous data are presented as medians with interquartile ranges (IQR) and compared between groups using Mann‐Whitney U‐tests. Correlations were analyzed using Pearson's correlation coefficient. All calculations were carried out using the IBM SPSS statistics package v21.

## 3 RESULTS

### 3.1 In vitro experiments using Er:YAG laser disinfection

**Figure 2:**
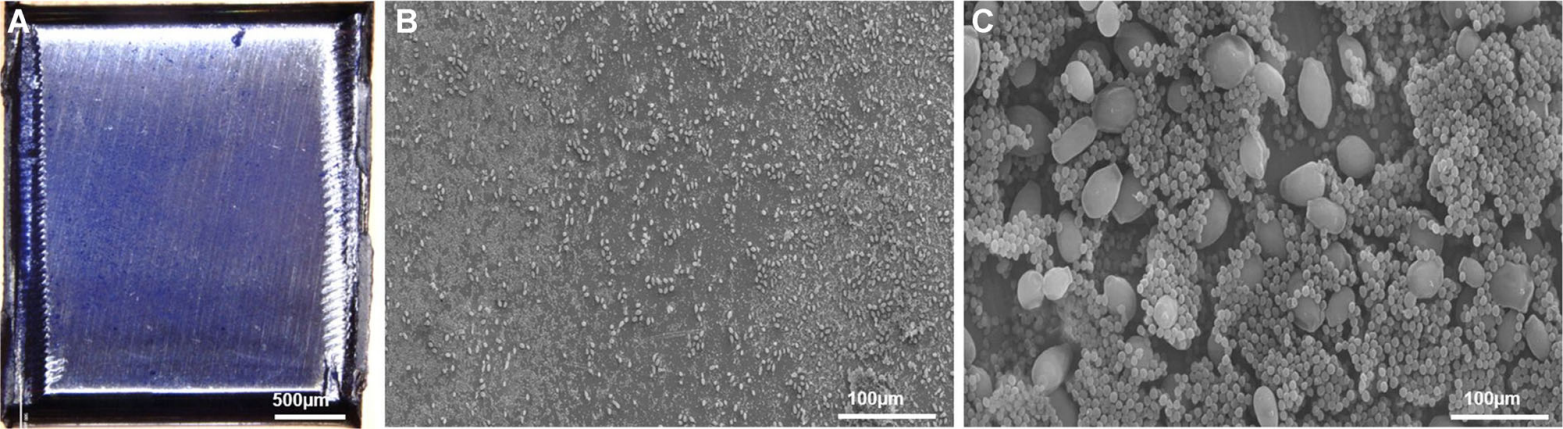
In vitro‐grown biofilm on steel plates. A, Macroscopic view of a crystal violet stained biofilm. B, Overview and C, detail SEM images showing several large yeast cells with bacteria in between. SEM, scanning electron microscopy [Color figure can be viewed at wileyonlinelibrary.com]

A series of 12 different laser energies (0.2‐2 J) was tested on the surface of in vitro‐grown biofilms on steel plates (Figure 2). Microbiology showed a reduction in vital bacteria by 99% and an elimination of all fungi already at pulse energies of 0.2 J (2.8 J/cm^2^). From the initial 1.75 × 10^6^ bacterial and 4.5 × 10^3^ fungal CFUs, 1.5 × 10^4^ bacterial CFUs and no viable fungi were present after treatment. A complete biofilm removal could be observed at all power settings from 0.3 J onwards.

**Figure 3:**
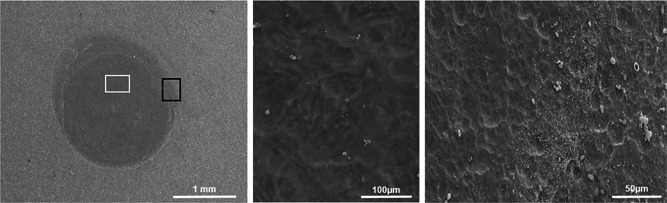
Determination of the minimally necessary laser energy for biofilm removal. Scanning electron micrographs of a cultivated metal plate after a single Er:YAG laser impulse show the discernible laser spots of 3 mm. Higher magnification reveals the complete removal of biofilm with 2 J. The very right images show the transition of the untreated biofilm and the lasered spot. A narrow transition area of homogenous residual material is found only in the periphery of the beam. Er:YAG, erbium:yttrium aluminum garnet

In SEM observations, the spots of the laser beam were clearly discernible and had sharp edges at the borders of the irradiated and the untreated biofilm. Inside the spot, the main part of the biofilm was removed with all settings tested. Residuals of the biofilm were found until 1.8 J. At 2 J, the surface was almost completely free of bacteria‐like structures (Figure 3). The steel surface of the plates did not show any structural changes.

**Figure 4:**
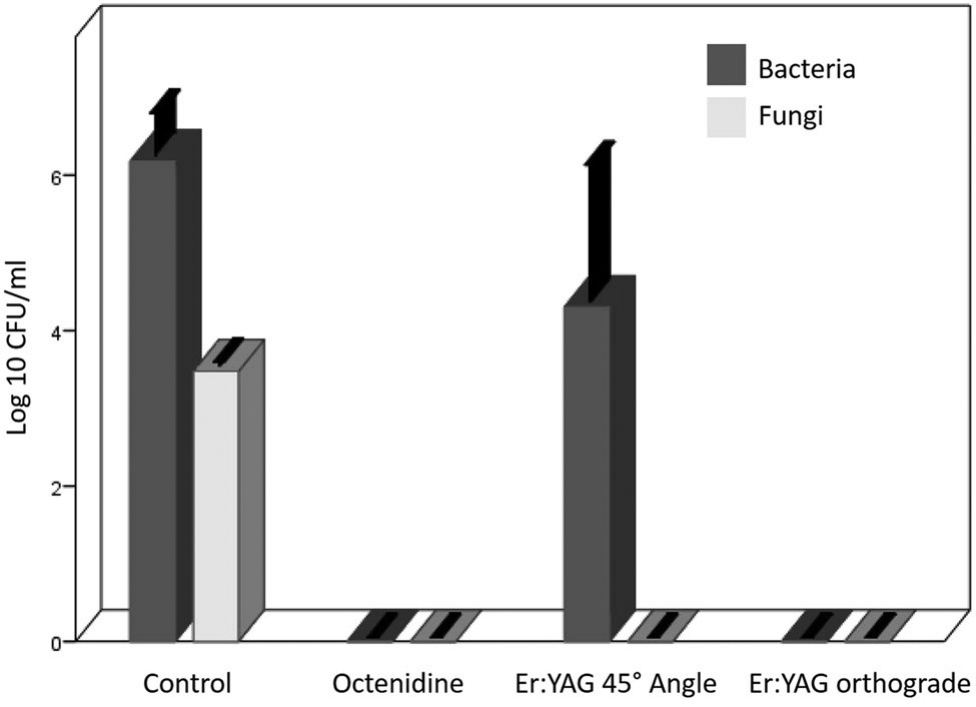
Comparison of the mean reduction in microbial contamination following different disinfection procedures in an in vitro model using cocultures. Median microbial colonization of in vitro‐cultivated pins (n = 42) after treatment. Whiskers indicate the 95% confidence intervals. Er:YAG, erbium:yttrium aluminum garnet

On the in vitro‐seeded pins, the biofilms in the control group contained an average of 1.96 × 10^6^ CFU of bacteria and 3 × 10^3^ CFU of yeast. Swab cleaning with octenidine achieved a complete eradication of bacterial and yeast colonization. Similarly, we did not see any remaining CFU after laser irradiation, when the pins were exposed to the laser beam (1.6 J) in a vertical manner. However, we found viable bacteria in two‐thirds of the pins when the laser beam struck the metal at a 45° angle (Figure 4).

**Figure 5:**
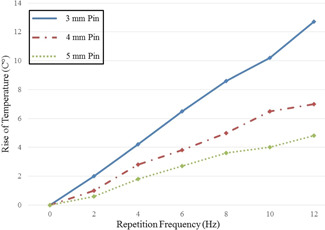
Temperature increase of steel pins after complete circumferential laser irradiation with 1.6 J. Pins of different diameter were analyzed at increasing laser frequencies [Color figure can be viewed at wileyonlinelibrary.com]

Because laser irradiation induces a temperature increase, pins of different diameters were measured with an infrared thermometer after single circumferential irradiation with different pulse energies and pulse repetition frequencies. As expected, higher repetition frequencies and pulse energies combined with small pin diameters lead to stronger heating. The highest temperature increase measured was +14.5°C in a 3 mm pin diameter at 2 J and 12 Hz (Figure 5).

**Figure 6:**
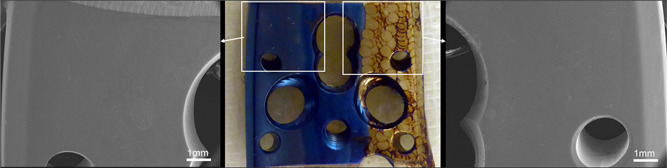
Titanium plates used for SEM examination were irradiated with the Er:YAG laser at two different power settings (22.8 J/cm^2^: middle third; 203 J/cm^2^: right third) or left untreated as control (left third). During the annealing process, no material is taken away, but a color change occurs through heating up the metal when high energy is used (right third). Despite this macroscopically visible change of color, neither SEM examination revealed a structural alteration of the titanium surface nor microbiology showed an altered bacterial opsonisation to the annealed surface. Er:YAG, erbium:yttrium aluminum garnet; SEM, scanning electron microscopy [Color figure can be viewed at wileyonlinelibrary.com]

During the examination of the titanium plates, annealing was only observed at very high‐power settings (>200 J/cm^2^), and we typically noted a change of the original blue color to brown, which is the first annealing color in order from lowest temperature to highest. However, the application of the Er:YAG laser with the previously described properties did not appear to have an effect on the microstructure or biocompatibility of the titanium surfaces (Figure 6). Particularly, we did not observe an increased biofim formation after annealing, since all plates were evenly seeded with microbes (S. aureus 5 × 10^3^ CFU and S. epidermidis 3 × 10^6^ CFU)

### 3.2 Ex vivo experiments using Er:YAG laser disinfection

We analyzed a total of 207 pins after their extraction from 43 external fixators from 41 patients. The external fixators were removed after a median time period of 7.3 weeks (IQR 3‐11). Seventy percent of pin sites were graded as “good,” 23% as “bad,” and 7% as “ugly” (Table 1).

**Table 1:** Characteristics of patients enrolled in this study

Patient	Age	Sex	Diagnosis	Number of Pins	Duration, wk	Clinical appearance^a^
1	36	M	Lux. gen. sin	4	3	1
2	5	M	Fract. fem. dext.	4	12	1
3	6	M	Fract. crur. dext.	4	8	1
4	56	M	Fract. rad. sin.	4	7	1
5	100	F	Luxfract. apert. bimall. dext.	3	3	2
6	43	M	Fract. apert. crur. sin.	7	6	1
7	63	M	Fract. crur. dext.	4	12	2
8	34	M	Fract. fem. dext.	8	3	3
9	34	M	Fract. apert. crur. dext.	11	9	1
10	46	M	Arthrodesis gen. sin.	8	12	2
11	15	M	Fract. fem. sin.	4	2	2
12	74	F	Fract. antebrach. sin.	4	8	1
13	31	F	Fract. crur. sin.	5	12	2
14	28	M	Fract. hum. dext.	4	3	2
15	67	F	Luxfract. bimall. dext	4	12	2
16	47	M	Fract. apert. crur. sin.	4	2	1
17	74	F	Fract. crur. dext. periprotetica	6	12	1
17^b^			Fract. crur. sin.	3	8	1
18	15	M	Fract. fem. dext.	4	3	1
19	30	M	Fract. pelvis.	4	10	3
20	63	F	Fract. phal. prox. dig. V. man. sin.	4	8	1
21	77	F	Fract rad. dist. dext.	4	12	1
22	60	F	Fract. apert. crur. sin.	5	11	1
23	68	F	Fract. rad. dist. sin.	4	8	1
24	32	M	Fract. crur. sin.	6	12	2
25	57	F	Fract. apert. antebrachii. dext	4	8	1
26	50	M	Fract. bimall. dext.	5	5	2
27	86	F	Fract. bimall. dext.	5	8	1
28	49	M	Fract. per‐ et subtroch. fem. dext	5	2	1
29	61	F	Fract. rad. dist. sin.	4	5	1
30	86	F	Fract. crur. dext.	6	16	1
31	14	F	Fract. tib. dext.	4	6	1
31^b^			Pilon tibiale	4	6	1
32	36	M	Fract. pertroch. fem. dext.	5	2	2
33	14	M	Fract. crur. dext	4	10	1
34	72	F	Pilon tibiale	3	5	1
35	14	M	Fract. crur. sin.	6	10	1
36	36	M	Pilon tibiale	3	13	1
37	47	M	Fract. apert. crur. dext.	6	5	1
38	42	M	Fract. Radii dist. dext.	4	3	1
39	58	M	Luxfract. apert. bimall. dext.	5	8	3
40	66	F	Luxfract. cubit. sin.	6	3	1
41	43	M	Fract. fem sin. et fract. crur. sin.	6	3	1
Mean	47.3			207.0	7.3	1.4
SD±	23.4			1.5	3.8	0.6

Abbreviation: SD±: standard deviation.

^a^ Clinical appearance according to the pin‐site grading system proposed by Clint et al.^32^

^b^ Patients treated with two external fixators.

Within the 20 different microbes identified in the biofilms from explanted pins, *S. epidermidis* was the most frequent bacteria associated with half pins (48.8%) and was detected in 41.6% of all patients showing signs of infection (pins graded “bad” or “ugly”). *Staphylococcus haemolyticus* and *S*. *aureus* were detected in 33.3% and 25% of all clinically infected patients, respectively.

The individual clinical assessment of the pins showed a significant correlation with the detected bacterial load identified (*r* = .398; *P* = .012). Pins rated as “good” showed a mean bacterial load of 6 CFUs (IQR 0‐77), those rated as “bad” 75 CFUs (IQR 11‐100), and those rated as “ugly” 181 CFUs (8‐350).

Comparing the results of swab cultures and sonication showed identical pathogens in 38% of our patients. In 33% of the patients, sonication detected additional bacteria that were not found in swab cultures. Vice versa, in 14%, the sonication was negative despite a positive swab culture. Additional pathogens of a mixed flora could be detected via sonication and via swab culture in 7.1% of cases. In 4.9% of patients included in this study, no microbes could be detected.

Microbes were considerably reduced after both, octenidine wiping, and laser irradiation. However, contrary to the in vitro experiments, detectable microbes via sonication were significantly less frequent in Er:YAG laser‐treated pins (2.5%) compared to octenidine‐treated pins (43.6%; *P* < .001) and the control group (79.5%; *P* < .001).

**Figure 7:**
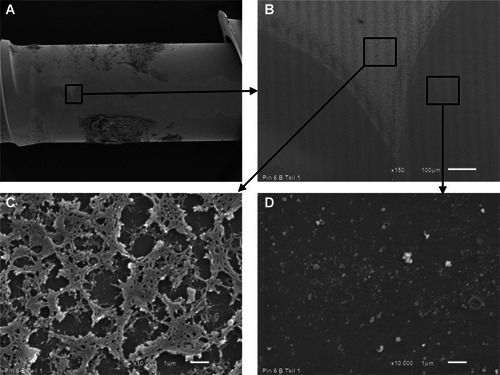
Scanning electron micrograph of an extracted pin after linear unidirectional irradiation with the Er:YAG laser. A, Overview picture with the trace of the laser beam horizontally in the middle. B, Two circular Er:YAG laser spots lying next to each other with sharp borders towards the adherent biofilm (magnification: ×150). C, High‐magnification view of biofilm noted in (B) (magnification: ×10 000). D, High‐magnification view of the laser irradiated area noted in (B) (magnification: ×10 000). Er:YAG, erbium:yttrium aluminum garnet

**Figure 8:**
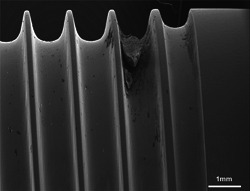
We found sporadic remnants of bacterial biofilms or debris in samples of the octenidine group

These quantitative data correspond with the SEM investigations which revealed that laser treatment removed the viable biofilm completely even if it was several 100 µm thick (Figure 7). Contrary, after octenidine wiping, sporadic nests of biofilm remained. Usually these nests were found in screw threads and other cavities (Figure 8).

## 4 DISCUSSION

In this study, the usefulness of the Er:YAG laser for the removal of biofilms from steel and titanium implants was tested with in vitro‐seeded as well as in vivo‐developed biofilms. The removal of bacteria and fungi was morphologically and microbiologically analyzed and compared with the state‐of‐the‐art treatment of mechanical cleaning with a liquid disinfectant. Furthermore, the spectrum of microbes forming biofilms on clinically used pins was analyzed and the efficacy of the harvesting methods (swabs and sonication) was assessed.

When investigating the appropriate laser settings, 0.3 J was identified as the lowest energy that completely devitalized the in vitro‐grown biofilm. In SEM, it was consistently observed that residuals of the biofilm were visible on the surface until 1.8 J, suggesting that not all of the devitalized biofilms were removed by this laser intensity. Almost complete clearance of the surface was observed at 2 J.

For comparison of Er:YAG laser irradiation (1.6 J, 22.8 J/cm^2^) with octenidine‐wipe disinfection of in vitro‐grown biofilms, both methods achieved a complete devitalization of all bacteria and fungi. In contrast, the examination of pins that were extracted from patients, showed a clear advantage of the Er:YAG laser treatment when compared to octenidine‐wipe disinfection. The different efficacy in removal of cultured and in vivo‐grown biofilms may be explained by a minor adherence or thickness of the cultivated biofilm that makes it more prone to removal by simple wiping than the thick biofilms grown within the human host with a certain amount of denatured proteins, inflammatory cells, and cellular debris. The residue‐free biofilm removal is of special concern when an infected implant is treated, since even a few remaining living cells can cause recurrent infections. We consider the ability of the Er:YAG laser for precise noncontact tissue removal and the induction of a sterile wound surface as a major advantage over other disinfection methods, particularly because peri‐implant soft tissue is of great importance in infection control.^1^, ^33^, ^34^, ^35^

The destructive effect of the Er:YAG laser derives from the spontaneous heating and evaporation of water that is part of every organism. However, heat is a double‐edged sword that has a distinct bactericidal or bacteriostatic effect but may also harm the surrounding tissue. If used in a clinical setting, some thermal damage to the surrounding tissue cannot be avoided (direct irradiation, reflection, and heating of the implant). It is difficult to say how much collateral thermal damage can be tolerated by the surrounding tissue without scarring, but according to Ross et al,^36^ it is not more than 300 µm in the perioral skin area. Even if skin is completely dissected with the Er:YAG laser, the thermal damage is restricted to a maximum of 210 µm, which makes scarring very unlikely.^37^ In this study, no pin was heated to temperatures critical for bone necrosis (50°C for 1 minute).^38^ Therefore, adverse effects on bone metabolism due to Er:YAG laser treatment are unlikely. In our setting, the temperature upshift of the implant was also not high (48°C) or long (10 min) enough to affect growth kinetics of the bacterial strains negatively.^39^ This is most probably the reason why the in vitro‐seeded microbes that were not directly hit by the laser beam at 45° inclination survived and were not damaged by transmitted heat. In addition, the loss of efficiency when using the laser in at 45° rather than perpendicular to the implant highlights the need for direct access to the surface of the implant. Anyhow, several cooling modalities (air/water) would be available if required, and a certain cooling affect can be expected from blood and adjacent tissue when clinically used.

Titanium is one of the so‐called reactive metals. This means that it reacts to certain conditions—current or heat, in this case—by developing only a few nanometers thin oxide layer that refracts light and creates the impression of color. In many industrial sectors (eg, medicine, aerospace), laser marking is used for titanium products as a nondestructive labeling method. Although macroscopically visible, the change of color at the titanium surface seems not to affect the biological properties of the implant.

To characterize the microflora in pin‐grown biofilms, samples were taken by swabs (before explanation) and sonication (of the extracted pins) from the same patients. *S. epidermidis, S. haemolyticus*, and *S*. *aureus* were the most common pathogens associated with clinically obvious pin infection. Therefore, an initial antibiotic therapy should address these pathogens. The advantages of sonication are widely accepted, especially in patients who had received antimicrobial therapy within 14 days before surgery.^31^, ^40^ Swab cultures should be avoided in the diagnosis of joint infection but still have their place in the diagnosis of infected pin sites.

The current study revealed the high‐potential of Er:YAG lasers for implant disinfection. However, certain limitations exist that need to be addressed. (a) In vitro‐ as well as in vivo‐grown biofilms are not completely regular, and these variations also influence the outcome. (b) Values may change with other sample sizes. (c) During temperature measurements, the baseline temperature was room temperature (22°C) rather than body temperature. (d) Due to the freehand nature of the laser treatment in this study, irregularities could have caused uneven irradiation of the implant surface. However, this circumstance simulates a possible clinical situation in which surgeons apply the laser freehand. The strength of this study design was that we could mimic a scenario as close as possible to a clinical setting using explanted pins from the same region and the same individual.

The implementation of an Er:YAG laser in a trauma care or orthopedic unit and the training are feasible. The acquisition costs are justified considering the possible avoidance of additional operative costs directly associated with the removal and replacement of infected pins calculated as $1330 per patient.^41^ Apart from the application in pin disinfection, laser cleaning could be helpful in even more challenging and cost‐intensive problems of implant and prosthetic joint infection.^42^, ^43^

Possible clinical application areas for the Er:YAG laser irradiation are early prosthetic joint infections or osteosynthesis‐associated infections that are treated with a debridement and retention concept. The laser irradiation during such operations can be carried out with relatively little time expenditure (~10 s/cm^2^). However, laser light can address only accessible regions of the implant and not the bone‐implant interface. Therefore, special laser handpieces would be auxiliary. We consider the combination of laser irradiation with a liquid disinfectant as a favorable modality in a clinical application.

## 5 CONCLUSION

This study showed that the Er:YAG laser allows simple and complete biofilm removal from metal implants without the risk of harming patient or implant. The method works regardless of which pathogens are involved. This is of particular importance because we are increasingly confronted with resistance of microbes to pharmaceutical and chemical substances. We consider the Er:YAG laser a precise noncontact biofilm removal strategy producing an antiseptic wound surface without any further tissue damage and without the risk of resistance of microbes. According to these properties, laser irradiation could become a valuable method in every DAIR procedure and should therefore be further investigated.

## CONFLICT OF INTERESTS

The authors declare that there are no conflict of interests.

## AUTHOR CONTRIBUTIONS

LKK: Experimental design, development of the indicated procedures, data acquisition, analysis and interpretation, statistical analysis, project funding by grant acquisition, and writing, drafting, and revising the manuscript. WH: Experimental design, contribution to the indicated procedures, acquisition, analysis and interpretation of data, final approval of the version to be published, and supervisory support. KD: Experimental design, data acquisition, analysis and interpretation, statistical analysis, and final approval of the version to be published. GT: Experimental design, data acquisition, analysis and interpretation, statistical analysis, and final approval of the version to be published. GK: Experimental design, data acquisition, analysis and interpretation, statistical analysis, and final approval of the version to be published. SK: Experimental design, data acquisition, analysis and interpretation, statistical analysis, and final approval of the version to be published. YT: Establishing the outpatient tests and analysis of the results and final approval of the version to be published. ML: Establishing the outpatient tests and analysis of the results and final approval of the version to be published. BZ: Establishing the outpatient tests and analysis of the results and final approval of the version to be published. EP: Experimental design, data acquisition, analysis and interpretation, statistical analysis, and final approval of the version to be published. SN: Experimental design, data acquisition, SEM investigation, analysis and interpretation, statistical analysis, project funding by grant acquisition manuscript writing, and final approval of the version to be published.

## References

[1] Zimmerli W , Trampuz A , Ochsner PE . Prosthetic‐joint infections. N Engl J Med. 2004;351(16):1645‐1654. 15483283 10.1056/NEJMra040181

[2] Gristina AG . Biomaterial‐centered infection: microbial adhesion versus tissue integration. Science. 1987;237(4822):1588‐1595. 3629258 10.1126/science.3629258

[3] Costerton JW , Stewart PS , Greenberg EP . Bacterial biofilms: a common cause of persistent infections. Science. 1999;284(5418):1318‐1322. 10334980 10.1126/science.284.5418.1318

[4] Ceri H , Olson ME , Stremick C , Read RR , Morck D , Buret A . The Calgary biofilm device: new technology for rapid determination of antibiotic susceptibilities of bacterial biofilms. J Clin Microbiol. 1999;37(6):1771‐1776. 10325322 10.1128/jcm.37.6.1771-1776.1999 PMC84946

[5] Anwar H , Costerton JW . Enhanced activity of combination of tobramycin and piperacillin for eradication of sessile biofilm cells of Pseudomonas aeruginosa. Antimicrob Agents Chemother. 1990;34(9):1666‐1671. 2126686 10.1128/aac.34.9.1666 PMC171902

[6] Costerton JW , Cheng KJ , Geesey GG , et al. Bacterial biofilms in nature and disease. Annu Rev Microbiol. 1987;41:435‐464. 3318676 10.1146/annurev.mi.41.100187.002251

[7] Khoury AE , Lam K , Ellis B , Costerton JW . Prevention and control of bacterial infections associated with medical devices. ASAIO J. 1992;38(3):M174‐M178. 1457842 10.1097/00002480-199207000-00013

[8] Masse A , Bruno A , Bosetti M , Biasibetti A , Cannas M , Gallinaro P . Prevention of pin track infection in external fixation with silver coated pins: clinical and microbiological results. J Biomed Mater Res. 2000;53(5):600‐604. 10984710 10.1002/1097-4636(200009)53:5<600::aid-jbm21>3.0.co;2-d

[9] Bosetti M , Masse A , Tobin E , Cannas M . Silver coated materials for external fixation devices: in vitro biocompatibility and genotoxicity. Biomaterials. 2002;23(3):887‐892. 11771707 10.1016/s0142-9612(01)00198-3

[10] Furno F , Morley KS , Wong B , et al. Silver nanoparticles and polymeric medical devices: a new approach to prevention of infection? J Antimicrob Chemother. 2004;54(6):1019‐1024. 15537697 10.1093/jac/dkh478

[11] Silver S . Bacterial silver resistance: molecular biology and uses and misuses of silver compounds. FEMS Microbiol Rev. 2003;27(2‐3):341‐353. 12829274 10.1016/S0168-6445(03)00047-0

[12] Lucke M , Schmidmaier G , Sadoni S , et al. Gentamicin coating of metallic implants reduces implant‐related osteomyelitis in rats. Bone. 2003;32(5):521‐531. 12753868 10.1016/s8756-3282(03)00050-4

[13] Voos K , Rosenberg B , Fagrhi M , Seligson D . Use of a tobramycin‐impregnated polymethylmethacrylate pin sleeve for the prevention of pin‐tract infection in goats. J Orthop Trauma. 1999;13(2):98‐101. 10052783 10.1097/00005131-199902000-00005

[14] Marotta JS , Coupe KJ , Milner R , Heseltine KE . Long‐term bactericidal properties of a gentamicin‐coated antimicrobial external fixation pin sleeve. J Bone Joint Surg Am. 2003;85‐A(suppl 4):129‐131. 10.2106/00004623-200300004-00017 14652404

[15] Jaskulka RA , Egkher E , Wielke B . Comparison of the mechanical performance of three types of unilateral, dynamizable external fixators. An experimental study. Arch Orthop Trauma Surg. 1994;113(5):271‐275. 7946818 10.1007/BF00443816

[16] Aro HT , Markel MD , Chao EY . Cortical bone reactions at the interface of external fixation half‐pins under different loading conditions. J Trauma. 1993;35(5):776‐785. 8230346 10.1097/00005373-199311000-00022

[17] Green SA . Complications of external skeletal fixation. Clin Orthop Relat Res. 1983;180:109‐116. 6627782

[18] Green SA , Ripley MJ . Chronic osteomyelitis in pin tracks. J Bone Joint Surg Am. 1984;66(7):1092‐1098. 6384221

[19] Mahan J , Seligson D , Henry SL , Hynes P , Dobbins J . Factors in pin tract infections. Orthopedics. 1991;14(3):305‐308. 2020629

[20] Maurer DJ , Merkow RL , Gustilo RB . Infection after intramedullary nailing of severe open tibial fractures initially treated with external fixation. J Bone Joint Surg Am. 1989;71(6):835‐838. 2745479

[21] Sims M , Saleh M . External fixation‐the incidence of pin site infection: a prospective audit. Orthop Nurs. 2000;4(2):59‐63.

[22] Walsh JT Jr. , Flotte TJ , Deutsch TF . Er:YAG laser ablation of tissue: effect of pulse duration and tissue type on thermal damage. Lasers Surg Med. 1989;9(4):314‐326. 2761327 10.1002/lsm.1900090403

[23] Hohenleutner U , Hohenleutner S , Baumler W , Landthaler M . Fast and effective skin ablation with an Er:YAG laser: determination of ablation rates and thermal damage zones. Lasers Surg Med. 1997;20(3):242‐247. 9138252 10.1002/(sici)1096-9101(1997)20:3<242::aid-lsm2>3.0.co;2-q

[24] Zipperle J , Schlimp CJ , Holnthoner W , et al. A novel coagulation assay incorporating adherent endothelial cells in thromboelastometry. Thromb Haemost. 2013;109(5):869‐877. 23494019 10.1160/TH12-10-0767

[25] Slee AM , O'Connor JR . In vitro antiplaque activity of octenidine dihydrochloride (WIN 41464‐2) against preformed plaques of selected oral plaque‐forming microorganisms. Antimicrob Agents Chemother. 1983;23(3):379‐384. 6847170 10.1128/aac.23.3.379 PMC184656

[26] Sedlock DM , Bailey DM . Microbicidal activity of octenidine hydrochloride, a new alkanediylbis[pyridine] germicidal agent. Antimicrob Agents Chemother. 1985;28(6):786‐790. 3909955 10.1128/aac.28.6.786 PMC180329

[27] Pitten FA , Werner HP , Kramer A . A standardized test to assess the impact of different organic challenges on the antimicrobial activity of antiseptics. J Hosp Infect. 2003;55(2):108‐115. 14529634 10.1016/s0195-6701(03)00260-3

[28] Amalaradjou MA , Norris CE , Venkitanarayanan K . Effect of octenidine hydrochloride on planktonic cells and biofilms of Listeria monocytogenes. Appl Environ Microbiol. 2009;75(12):4089‐4092. 19376913 10.1128/AEM.02807-08 PMC2698330

[29] Park YJ , Song HJ , Kim I , Yang HS . Surface characteristics and bioactivity of oxide film on titanium metal formed by thermal oxidation. J Mater Sci Mater Med. 2007;18(4):565‐575. 17546415 10.1007/s10856-007-2303-7

[30] Hwang KS , Lee YH , Kang BA , Kim SB , Oh JS . Effect of annealing titanium on in vitro bioactivity. J Mater Sci Mater Med. 2003;14(6):521‐529. 15348436 10.1023/a:1023408030405

[31] Trampuz A , Piper KE , Jacobson MJ , et al. Sonication of removed hip and knee prostheses for diagnosis of infection. N Engl J Med. 2007;357(7):654‐663. 17699815 10.1056/NEJMoa061588

[32] Clint SA , Eastwood DM , Chasseaud M , Calder PR , Marsh DR . The "good, bad and ugly" pin site grading system: a reliable and memorable method for documenting and monitoring ring fixator pin sites. Injury. 2010;41(2):147‐150. 19647820 10.1016/j.injury.2009.07.001

[33] Tande AJ , Patel R . Prosthetic joint infection. Clin Microbiol Rev. 2014;27(2):302‐345. 24696437 10.1128/CMR.00111-13 PMC3993098

[34] McConoughey SJ , Howlin R , Granger JF , et al. Biofilms in periprosthetic orthopedic infections. Future Microbiol. 2014;9(8):987‐1007. 25302955 10.2217/fmb.14.64 PMC4407677

[35] Neut D , van der Mei HC , Bulstra SK , Busscher HJ . The role of small‐colony variants in failure to diagnose and treat biofilm infections in orthopedics. Acta Orthop. 2007;78(3):299‐308. 17611841 10.1080/17453670710013843

[36] Ross EV , McKinlay JR , Anderson RR . Why does carbon dioxide resurfacing work? A review. Arch Dermatol. 1999;135(4):444‐454. 10206052 10.1001/archderm.135.4.444

[37] Kriechbaumer LK , Susani M , Kircher SG , Happak W . Vaporization of cutaneous neurofibromas with an erbium: yttrium‐aluminum‐garnet laser: a comparative histologic evaluation. Plast Reconstr Surg. 2012;129(3):602e‐604e. 10.1097/PRS.0b013e3182419d22 22374043

[38] Eriksson RA , Albrektsson T , Magnusson B . Assessment of bone viability after heat trauma. A histological, histochemical and vital microscopic study in the rabbit. Scand J Plast Reconstr Surg. 1984;18(3):261‐268. 6549359 10.3109/02844318409052849

[39] Fleury B , Kelley WL , Lew D , Gotz F , Proctor RA , Vaudaux P . Transcriptomic and metabolic responses of Staphylococcus aureus exposed to supra‐physiological temperatures. BMC Microbiol. 2009;9:76. 19386094 10.1186/1471-2180-9-76 PMC2687450

[40] Esteban J , Alonso‐Rodriguez N , del‐Prado G , et al. PCR‐hybridization after sonication improves diagnosis of implant‐related infection. Acta Orthop. 2012;83(3):299‐304. 22616742 10.3109/17453674.2012.693019 PMC3369159

[41] Coyte PC , Bronskill SE , Hirji ZZ , Daigle‐Takacs G , Trerise BS , Wright JG . Economic evaluation of 2 treatments for pediatric femoral shaft fractures. Clin Orthop Relat Res. 1997;336:205‐215. 10.1097/00003086-199703000-00029 9060507

[42] Peel TN , Dowsey MM , Buising KL , Liew D , Choong PF . Cost analysis of debridement and retention for management of prosthetic joint infection. Clin Microbiol Infect. 2013;19(2):181‐186. 22264335 10.1111/j.1469-0691.2011.03758.x

[43] Klouche S , Sariali E , Mamoudy P . Total hip arthroplasty revision due to infection: a cost analysis approach. Orthop Traumatol, Surg Res. 2010;96(2):124‐132. 20417910 10.1016/j.rcot.2010.02.005

